# Study of active components and mechanisms mediating the hypolipidemic effect of *Inonotus obliquus* polysaccharides

**DOI:** 10.1002/fsn3.3964

**Published:** 2024-02-06

**Authors:** Guanwen Ding, Xiao Guo, Xiao Li, Liping An, Huawen Shi

**Affiliations:** ^1^ Clinical Medical College Harbin Medical University Harbin China; ^2^ School of Pharmacy Beihua University Jilin China; ^3^ Harbin Medical University Cancer Hospital Harbin China

**Keywords:** hyperlipidemia, hypolipidemic, *Inonotus obliquus* polysaccharide, isolation and purification

## Abstract

Hyperlipidemia is a multifaceted metabolic disease, which is the major risk factor for atherosclerosis and cardiovascular diseases. Traditional Chinese medicine provides valuable therapeutic strategies in the treatment of hyperlipidemia. *Inonotus obliquus* has been used in traditional medicine to treat numerous diseases for a long time. To screen and isolate the fractions of *I. obliquus* polysaccharides (IOP) that can reduce blood lipid in the hyperlipemia animals and cell models, and investigate its mechanisms. The active component IOP‐A2 was isolated, purified, and identified. In vivo, rats were randomly divided into blank control group (NG), the high‐fat treatment group (MG), lovastatin group (PG), and IOP‐A group. Compared with MG, the hyperlipidemic rats treated with IOP‐A2 had decreased body weight and organ indexes, with the level of serum total cholesterol (TC), triglyceride (TG), and low‐density lipoprotein cholesterol (LDL‐C) significantly decreased (*p* < .05), and level of serum high‐density lipoprotein cholesterol (HDL‐C) significantly increased (*p* < .05). Hepatocyte steatosis in hepatic lobules was significantly reduced. In vitro, the accumulation of lipid droplets in the model of fatty degeneration of HepG2 cells was significantly alleviated, and cellular TC and TG content was significantly decreased (*p* < .01). Moreover, the expression of recombinant cytochrome P450 7A1 (CYP7A1) and Liver X Receptor α (LXRα) were up‐regulated (*p* < .05) both in vivo and in vitro. The results showed that IOP‐A2 may exert its hypolipidemic activity by promoting cholesterol metabolism and regulating the expression of the cholesterol metabolism‐related proteins CYP7A1, LXRα, SR‐B1, and ABCA1.

## Graphical abstract

Hyperlipidemia is a kind of multifaceted metabolic disease, which is the major risk factor for atherosclerosis and cardiovascular diseases. Traditional Chinese medicine provide valuable therapeutic strategies in the treatment of hyperlipidemia.

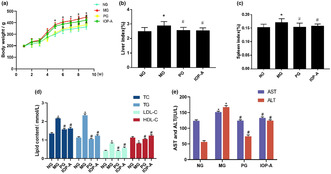

## 1 INTRODUCTION

Hyperlipidemia is a common and multi‐faceted metabolic disease that involves abnormally high levels of various lipids. It characterizes by abnormal increase in cholesterol, triglycerides, and lipoprotein level, and a series of clinical and pathological manifestations that severely affect human health (Lv et al., 2019; Woo, 2018). The most important and direct damage caused by hyperlipidemia is the acceleration of systemic atherosclerosis, which often affects arteries in more than one location and closely related to stroke, diabetes, hypertension, fatty liver, and other diseases (Shao et al., 2017; Zhang et al., 2021). It is reported that plasma cholesterol is a pathogenic risk factor for coronary heart disease. Active treatment of hyperlipidemia can significantly reduce the disability and mortality of coronary heart disease (Navar‐Boggan et al., 2015). At present, there are many clinical treatments, including statins, fibrates, and other lipid‐lowering drugs, and their long‐term use may produce some negative side effects. Chinese herbal medicines have small toxic effects and reduce damage to the liver and kidneys. Therefore, it is widely used in the treatment of diseases (Li et al., 2022). In the treatment of hyperlipidemia, many herbal medicines (including herbal formulas, natural extracts, and pure bioactive compounds from medicinal plants) (Han et al., 2019; Lin et al., 2016; Park et al., 2019), such as red ginseng polysaccharides, Ganoderma lucidum polysaccharides, curcumin, etc., have been proven to inhibit liver adipogenesis by reducing the expression of key transcription factors and lipogenic enzymes, thus regulating the disorder of lipid metabolism. (Kwak et al., 2010; Li, Zhao, et al., 2020; Panahi et al., 2017) As a result, the primary focus in this field has shifted to the development of new drugs derived from natural active ingredients with significant lipid‐regulating effects and minor side effects.

Fungal polysaccharides, which are natural and safe hypolipidemic substances, are medicinal active components found at high levels in various edible fungi (Kim et al., 2020). *Inonotus obliquus*, also known as birch mushroom, white birch antler, or birch antler, parasitizes damaged trunks or bark of birch, elm, and red poplar, among other tree species, and is one of the ten most precious medicinal fungi in the world (Yu et al., 2021). *Inonotus obliquus* polysaccharides (IOP) are one of the primary active ingredients and possess several pharmacological activities, including anti‐cancer, anti‐inflammatory, anti‐oxidant, hypoglycemic, and immuno‐stimulatory properties (Jiang et al., 2020; Xu et al., 2019). The polysaccharide has a complex structure and a high degree of polymerization and can be composed of more than 10 identical or different monosaccharides linked by α‐ or β‐glycosidic bonds. Its molecular weight can reach tens of thousands or even millions of Da (Duru et al., 2019). The chemical composition, structure, and conformation of polysaccharides may determine their biological activities to a great extent (Yang & Zhang, 2009; Zhang, Wen, et al., 2019). Recently, study on the function of fungal polysaccharides becomes a hot area of modern medicine and food functional chemistry research. Xue found that IOEP1 (*I. obliquus* hypoglycaemic activities of the polysaccharides) and IOEP2 (*I. obliquus* hypoglycaemic activities of the polysaccharides) strongly increased HepG2 cell's glucose consumption and insulin‐resistant HepG2 cells, which proved that IOEP1 and IOEP2 might be suitable anti‐diabetes agents in functional foods and natural drugs (Xue et al., 2018). Wang studied the antidiabetic effect of IOPS and its underlying mechanism in vivo and found that the possible hypolipidemic mechanism may be through the regulation of PI3K/Akt (Phosphatidylinositol 3 kinase/protein kinase B) and AMPK/ACC (AMP‐activated protein kinase/acetyl‐CoA carboxylase) signaling pathways (Wang et al., 2017). Although the development of IOPS has made some progress in the past decade, there are still some problems that cannot be ignored. On the one hand, it is of substantial consequence to delve into the underlying mechanism of its biological activity. On the other hand, previous studies lacked reproducibility, so it is of great significance for us to further study the lipid‐lowering active components of IOP and its mechanism (Xu et al., 2019; Yang et al., 2021).

In this study, we isolated, purified, and identified *I. obliquus* acidic polysaccharides (IOP‐A). By establishing a high‐fat diet rat model and oleic acid induced high lipid load HepG2 cell model, we observed the hypolipidemic effect of IOP‐A and explored the mechanism of hypolipidemic effect of IOP‐A in order to provide a scientific basis for the further development of related functional drugs for IOP.

## 2 EXPERIMENTAL MATERIALS

### 2.1 Animal and cell

Four‐ to five‐week‐old specific‐pathogen‐free (SPF) male Sprague Dawley (SD) rats weighing 190.0 ± 2.0 g were purchased from Changchun Yisi Experimental Animal Technology Co., Ltd. (Changchun, China; License number: SCXK (Ji)‐2016–0003). Animal experiments were approved by the Institutional Animal Care and Use Committee (IACUC) of Beihua University. All experimental procedures were carried out in accordance with the Guidelines on the Care and Use of Laboratory Animals (China).

The human hepatoma cell line HepG2 was purchased from the Cell Bank of Peking Union Medical College Hospital (Beijing, China).

### 2.2 Materials, reagents, and Main instrument

*Inonotus obliquus* (Jiangsu Suwei Micro‐biology Research Co., Ltd., Jiangsu, China) was identified as the dry fruiting body of *I. obliquus* by the Department of Pharmacognosy, College of Pharmacy, Beihua University, China. DEAE cellulose, Sepharose C‐6Bs (Sigma Aldrich, USA); RIPA lysis buffer (Beijing Dingguo Changsheng Biotechnology Co., Ltd, Beijing, China); Caihong marker (GE Healthcare, USA); anti‐CYP7A1, ‐LXRα, ‐SR‐B1, ‐ABCA1, and ‐β‐actin monoclonal antibodies (Abcam, UK); goat anti‐rabbit IgG labeled with horseradish peroxidase (Abcam, UK); total cholesterol kit (TC), triglyceride kit (TG), high‐ and low‐density lipoprotein cholesterol (HDL‐C, LDL‐C) kit (Zhejiang Tianhang Biotechnology Co., Ltd., Zhejiang, China); aspartate aminotransferase (AST) and alanine aminotransferase (ALT) kit (Wako Pure Chemical Industries Ltd., Osaka, Japan); RPMI‐1640 (Hyclone, USA); trypsin (GENVIEW, USA); MTT (Sigma Aldrich, USA); oil red O (Salarbio Life Sciences, Beijing, China); BCA protein quantitative determination kit (Beijing Dingguo Changsheng Biotechnology Co., Ltd, Beijing, China). All the other reagents had purity of analytical grade. Shimadzu HPLC liquid chromatograph (Shimadzu International Trade Co., Ltd., Japan); gas chromatograph (Agilent Technology Co., Ltd., China); nuclear magnetic resonance spectrometer (Bruker AVANCE, Beijing, China); UV‐2550 ultraviolet visible spectrophotometer (Shimadzu International Trade Co., Ltd., Japan); infinite M200 enzyme labelling apparatus (TECAN, USA) electrophoresis apparatus (Bio‐rad Company, USA); fully automatic fluorescence and chemiluminescence gel imager (Beijing Sage Creation Technology Co., Ltd., China); cell incubator (Sanyo MCO‐19AIC, Japan).

## 3 METHODS

### 3.1 Extraction and isolation of IOP

Inonotus obliquus was soaked in deionized water at a solid–liquid ratio of 1: 20 (w/v) overnight, and extracted by hot reflux extraction at 90°C for 2 h, repeated three times. Then, the extract was filtered and mixed, concentrated at 60°C under vacuum. Four times the volume of anhydrous ethanol was added to the concentrated solution, and the mixture was incubated overnight at 4°C. The next day, the mixture was centrifuged (4000 rpm for 10 min), and the pellet was collected. After two successive washes with respectively 95% ethanol and anhydrous ethanol, the precipitates were collected and dried routinely to obtain the crude IOP. The solution was dialyzed using a dialysis bag with a molecular weight of 3500 Da, and the dialysis extract was concentrated under reduced pressure and dried and frozen to obtain a purified IOP water extract (Li, Lu, et al., 2020; Xu et al., 2019).

### 3.2 Purification of IOP

IOP was dissolved in distilled water and fractionated on a DEAE‐cellulose column chromatography (7.5 × 30 cm, Cl‐Type). The elution used distilled water and 0.5 M NaCl solution at a flow rate of 2 mL/min. The eluted polysaccharide was quantified by the phenol‐sulfuric acid method (Dubois, M., Giles, K., Rebers, P.et al. 1956). The resulting catalysis generated a neutral *I. obliquus* polysaccharide (IOP‐N, distilled water) and an *I. obliquus* acidic polysaccharide (IOP‐A, 0.5 M NaCl). The IOP‐A obtained by 0.5 M NaCl elution was further separated and purified on a Sepharose Cl‐6B column (2.6 × 100 cm) using a 0.15 M NaCl solution for elution at a flow rate of 0.4 mL/min (Luo, 2008). Compared with IOP‐N, IOP‐A was chosen with high activities based on pre‐experiments. So using this method, large quantities of IOP‐A were prepared for the subsequent experiments (Wang et al., 2021).

### 3.3 Identification of IOP‐A2

#### 3.3.1 Chemical composition analysis

The total polysaccharide content of each sample was determined by phenol sulfuric acid with glucose as standard (Blumenkrantz & Asboe‐Hansen, 1973). The uronic acid content of the samples was determined by the M‐hydroxyphenyl method, using D‐galacturonic acid as standard (Bennett & Scott, 1971). The protein content of the samples was determined by coomassie brilliant blue method, using bovine serum albumin as standard (Kwak et al., 2010).

#### 3.3.2 Determination of the molecular weights

Two milligrams of IOP‐A2 sample were dissolved in 0.2 M NaCl to a final concentration of 5 mg/mL. The solution was filtered through a 0.22 μm filter membrane, and 20 μL of the filtrate was analyzed by high‐performance gel permeation chromatography (HPGPC), using a LC‐10Avp system (Shimadzu Company) with a RID‐10A differential refraction detector and a TSK‐gel G‐3000 PWXL column (7.8 × 300 mm). The column temperature was 35°C, and the mobile phase was a 0.2 M NaCl solution used at a flow rate of 0.6 mL/min. A series of dextran with different molecular weights (1 kDa, 5 kDa, 12 kDa, 25 kDa, 50 kDa) was used to calibrate the chromatographic column and built a standard curve (Zhang, Liu, et al., 2019).

#### 3.3.3 Monosaccharide composition analysis

Two milligrams of IOP‐A2 were dissolved in 1 mL of 2 M HCl/methanol and hydrolyzed at 80°C for 8 h. The sample was further hydrolyzed with 1 mL of 2 M trifluoroacetic acid (TFA) at 120°C for 1 h. The hydrolysate was derivatized with 1‐phenyl‐3‐methyl‐5‐pyrazolone (PMP) prior to the application of a DIKMA Inertsil ODS‐3 column (4.6 mm × 150 mm) and analyzed with a Shimadzu HPLC system (LC‐10ATvp pump and SPD‐10AVD UV–VIS detector). The monosaccharide composition was determined and quantified by taking the retention time and peak area of a standard monosaccharide as reference. (Needs & Selvendran, 2010).

#### 3.3.4 Methylation analysis

A modified method (Lee et al., 2019; Li et al., 2014) was adopted for methylation analysis. All the hydroxyl (‐OH) groups of the sample were considered completely methylated if no obvious absorption peak was detectable at 3400 cm^−1^ in the Fourier‐transform infrared (FT‐IR) spectrum. Then, the methylated polysaccharide was hydrolyzed, reduced, and acetylated, and the final product was analyzed by gas chromatography–mass spectrometry (GC–MS) with an Agilent Technologies 7890b‐5977B instrument. The heating procedure was as follows: 120°C for 1 min; 210°C for 2 min (heating rate: 3°C/min); 260°C for 4 min (heating rate: 10°C/min). Inositol was used as an internal reference. Partially methylated aldosterone acetate was identified according to its retention time and fragmentation mode.

#### 3.3.5 NMR analysis

20 mg of dried IOP‐A2 were dissolved in 0.5 mL D_2_O (99.8%), and the ^13^C‐NMR spectrum of the sample was determined with a Bruker Avance 600 MHz spectrometers (Germany) at 20°C, using a detection frequency of 150 MHz. (Barb et al., 2011).

### 3.4 Establishment of a rat hyperlipidemia model and assessment of treatment effect in vivo

Thirty‐two SPF‐grade male SD rats, aged 4–5 weeks, were acclimatized to the laboratory environment for 1 week and then randomly divided into a blank control group (NG), a model group (MG), a lovastatin group (PG) and IOP‐A group, each containing eight rats. The animals in the NG were fed a basal diet (100 g/kg/day), whereas those in the other three groups were fed a high‐fat diet (100 g/kg/day). In addition, the PG rats were given lovastatin (8.4 mg/kg/day), and those in IOP‐A2 group were given IOP‐A 450 mg/kg/day, which was screened from 100 mg/kg/day to 900 mg/kg/day in a pre‐experimental. All rats could drink freely in the environment of a humidity level of 50%–60% and a temperature of 20.0°C–22.0°C. High‐fat diet feeding and the different treatments lasted for 8 weeks. (Saiki et al., 2007).

### 3.5 Calculation of body weight and organ indexes

The rats were weighed once a week during the feeding period. Eight weeks later, the spleen and liver were collected, and washed with normal saline, and their wet weight were weighed. (Navar‐Boggan et al., 2015). The spleen and liver indexes were calculated as follows:

$$\text{Organ index}=\text{organ mass}\;\left(\mathrm{mg}\right)/\text{body weight}\;\left(g\right)\times 100\%.$$

### 3.6 Histological analysis

Hematoxylin and eosin (HE) staining was performed for histopathological analysis. Liver samples that were fixed in 10% buffered formalin and paraffin‐embedded were sliced into 4 μm‐thick sections and stained with HE. The morpho‐pathological changes resulting from the high‐fat diet, and the different treatments were analyzed under an optical microscope. (Kwon et al., 2018).

### 3.7 Cell culture

After thawing, cryopreserved HepG2 cells were cultured in RPMI 1640 medium containing 10% fetal bovine serum at 37°C and under 5% CO_2_ with 90% humidity in a cell incubator. (Alnahdi et al., 2019).

### 3.8 Screening of IOP active components

HepG2 cells in the logarithmic growth phase were cultured in a 6‐well plate for 48 h, induced by 0.5 mmol/L OA for 24 h and then treated with 5 μg/mL of either IOP‐A1, IOP‐A2, or IOP‐A3 for 24 h. After treatment, the cells were collected, and the TC and TG levels were determined according to the instructions provided in the kits, in order to screen for the active components of IOP. (Tao et al., 2021).

### 3.9 Detection of cell proliferation with the MTT method

HepG2 cells were seeded in 96‐well plates at a density of 1 × 10^5^/well, and after plating for 16 h, IOP‐A at 0.625, 1.25, 2.5, 5, 10 μg/mL was added to the plate. Cells were cultured for 24 h to measure cell viability using an MTT solution as described (Liou et al., 2019). The absorbance value of cells in each group at 540 nm was detected by spectrophotometer.

### 3.10 In vitro treatment and IOP‐A2 dosage

IOP‐A2 was selected for further analysis in vitro. HepG2 cells were split into five groups: a normal control group (NG), model group (MG, 0.5 mmol/L oleic acid [OA]), low‐dose IOP‐A2 group (IOP‐A2‐L, 0.5 mmol/L OA + 2.5 μg/mL IOP‐A2), medium‐dose IOP‐A2 group (IOP‐A2‐M, 0.5 mmol/L OA + 5 μg/mL IOP‐A2), and high‐dose IOP‐A2 group (IOP‐A2‐H, 0.5 mmol/L OA + 10 μg/mL IOP‐A2) (Norouzzadeh et al., 2016).

### 3.11 Oil red O staining

HepG2 cells in the logarithmic growth phase were cultured in 6‐well plates for 48 h, then induced by 0.5 mmol/L oleic acid for 24 h and treated with the different doses of IOP‐A2 for another 24 h. Then, the culture medium was discarded. The cells were washed with PBS three times, fixed in 4% paraformaldehyde for 20 min, washed with PBS three times, mordanted with 60% isopropanol for 15 s, and stained with oil red O for 30 min. After staining, the cells were washed with PBS three times, differentiated with 60% isopropanol for 10 s, and washed again with PBS three times. The accumulation of lipid droplets in the cells was evaluated under an inverted microscope (Lee et al., 2011).

### 3.12 Determination of biochemical indexes in the experimental animals and cell cultures

The blood of the rat was collected from the abdominal aorta and centrifuged at 3500 rpm for 15 min to separate the serum. The serum was stored at −20°C until further use. Serum TC, TG, LDL‐C, HDL‐C, AST, and ALT levels were determined according to the manufacturer's instructions provided in the kits.

### 3.13 Detection of expression of CYP7A1, LXRα, SR‐B1 and ABCA1 proteins by western blot

Protein extracts from rat livers and cultured cells were prepared by the standard method (Yang et al., 2019). The protein concentration was determined by BCA method using a standard curve with a reference protein and equalized between samples. For the Western blot, a volume of 20 μL of protein sample was loaded per well, and the proteins were separated by SDS‐PAGE electrophoresis. The separated proteins were transferred onto a PVDF membrane. The membrane was incubated in a blocking buffer containing 5% skim milk powder under horizontal agitation at room temperature for 2 h. The membranes were washed with TBST, then diluted primary antibodies (anti‐CYP7A1, ‐LXRα, ‐SR‐B1, ‐ABCA1 and ‐β‐actin) were added to the membranes and incubated at 4°C overnight. Secondary anti‐rabbit immunoglobulins were added onto the membranes, and the membranes were incubated at room temperature for 1 h, washed, and incubated with an ECL luminescent solution. For quantitative analysis, the images were developed and photographed with a gel image.

### 3.14 Statistical methods

All values are expressed as mean ± standard deviation (mean ± s). SPSS software was used for statistical analysis. *p* < .05 or *p* < .01 were considered statistically significant.

## 4 RESULTS

### 4.1 Preparation of IOP‐A2

**FIGURE 1:**
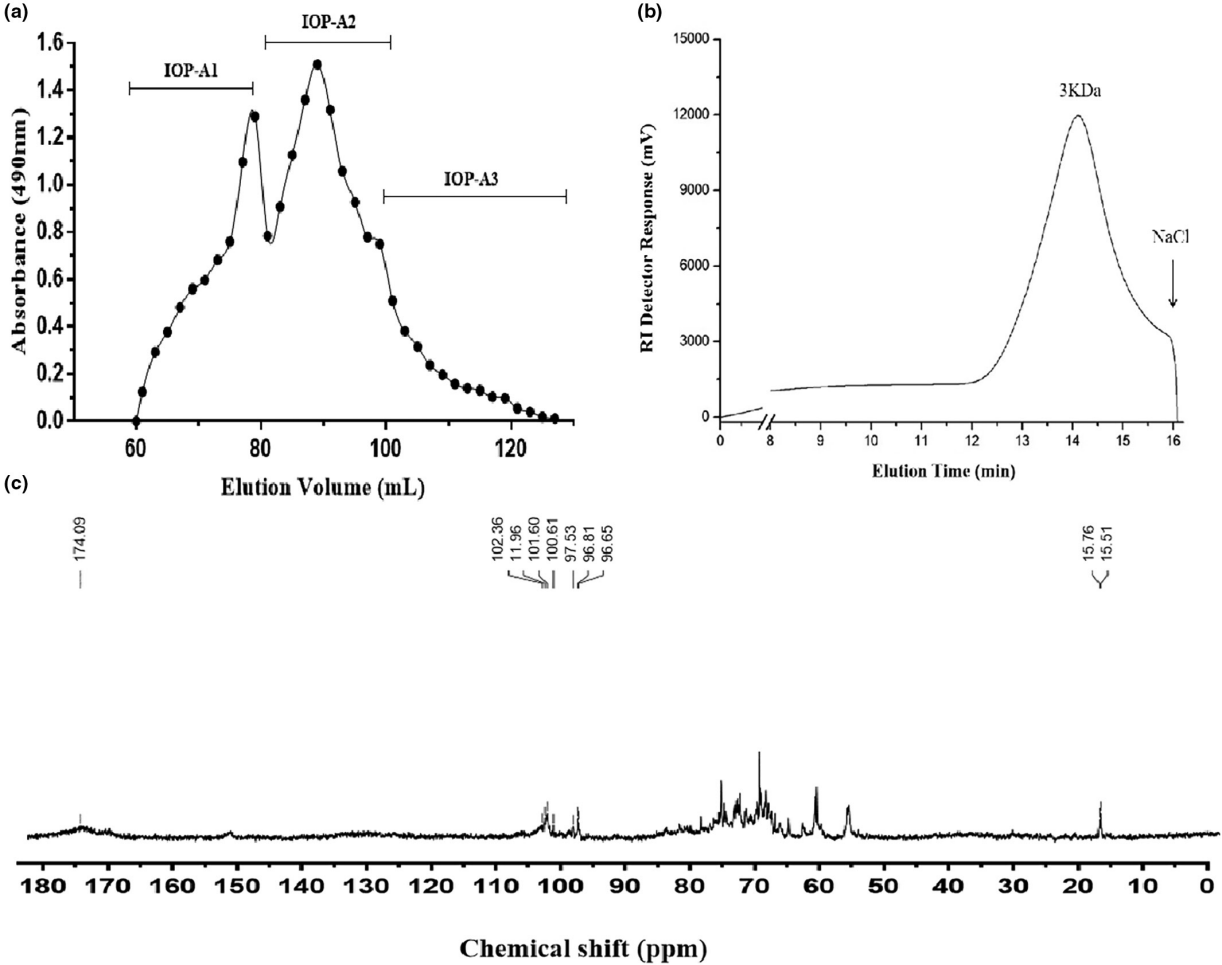
(a) Gel chromatography elution curve of IOP‐A fractions; (b) HPGPC elution curve of IOP‐A2; (c) ^13^C NMR spectrum of IOP‐A2.

A crude IOP extract was obtained from *I. obliquus* with a yield of 8.4%. A gradient elution of this IOP extract by DEAE cellulose ion exchanges column chromatography revealed that IOP was composed of a neutral polysaccharide fraction IOP‐N (34.5%), and an acidic polysaccharide fraction IOP‐A (43.8%). The IOP‐A was further fractionated on a Sepharose‐CL‐6B column. After freeze‐drying of the eluates, three sub‐fractions—IOP‐A1, IOP‐A2, and IOP‐A3—were obtained according to the HPGPC analysis (Figure 1a), with yields of 20.1%, 39.3%, and 16.8%, respectively. IOP‐A2, which presented with the highest hypolipidemic activity, was selected for further investigation.

### 4.2 Monosaccharide composition and structural features of IOP‐A2

The homogeneity of the above polysaccharide fractions was probed by HPGPC. The molecular weight of IOP‐A2 was 3 kDa (Figure 1b), and the HPLC analysis showed that IOP‐A2 was mainly composed of Gal (24.38%), Glc (24.22%), GalA (14.47%), Man (14.08%), Rha (6.76%), Xyl (5.32%), GlcA (4.91%), Fuc (0.71%), and Ara (0.14%).

The glycosidic linkages of polysaccharides were determined by methylation and GC–MS analysis. The list of glycosylations encountered in fraction IOP‐A2 is shown in Table 1. The main linkages in IOP‐A2 were 1,6‐Gl*cp*, 1,3‐Gl*cp*, 1,3,6‐Gal*p* and 1,2‐Man*p* (10.59%, 8.12%, 12.60%, and 9.95%, respectively), and there were also few other types of linkage.

**TABLE 1:** Methylation analysis of IOP‐A2 by NMR spectroscopy.

Sample	Methylation types	Linkage	Percentage	Fragment ions (m/z)
IOP‐A2	2,3,4,6‐Me_4_‐Glc*p*	1‐	3.22	101,117,129,145,161,205
	2,3,6‐Me_3_‐Glc*p*	1,4‐	1.18	87,101,117,129,233
	2,3,4‐Me_3_‐Glc*p*	1,6‐	10.59	87,101,117,127,261
	2,4,6‐Me_4_‐Glc*p*	1,3‐	8.12	101,117,129,145,161,205
	2,4‐Me_4_‐Glc*p*	1,3,6‐	5.28	87,117,129,139,159,189,233
	2,3,4,6‐Me_4_‐Gal*p*	1‐	3.31	87,101,117,129,145,161,205
	2,4,6‐Me_3_‐Gal*p*	1,3‐	2.34	101,117,129,161,233
	2,3,4‐Me_3_‐Gal*p*	1,6‐	7.99	87,101,117,129,161,173,189,233
	2,3,6‐Me_3_‐Gal*p*	1,4‐	1.69	87,101,117,129,161,175,233
	2,4‐Me_2_‐Gal*p*	1,3,6‐	12.60	87,117,129,139,159,189,233
	2,6‐Me_2_‐Man*p*	1,3,4‐	3.61	87,117,129,143,185,203
	2,3,4,6‐Me_4_‐Man*p*	1‐	2.62	87,101,117,129,145,161,205
	3,4,6‐Me_3_‐Man*p*	1,2‐	9.95	87,129,161,189
	2,3,4‐Me_3_‐Rha*p*	1‐	0.82	89,101,117,131,161
	3,4‐Me_2_‐Rha*p*	1,2‐	3.92	89,131,189
	2,4‐Me_2_‐Rha*p*	1,3‐	1.47	87,101,117,129,189
	3‐Me‐Rha*p*	1,2,4‐	1.73	87,101,129,143,189,205
	2,3,4‐Me_3_‐Xyl	1,6‐	2.74	87,101,117,161,189,233
	2,4‐Me_2_‐Xyl	1,2‐	4.21	87,131,161,173,205
	2,3,5‐Me_3_‐Ara*f*	1‐	0.76	87,101,117,129,161
	2,3‐Me‐Ara*f*	1,5‐	0.45	87,99,117,129,189,207

^13^C NMR spectral analysis can display the anomeric carbon signal and chemical shift of IOP‐A2 with a wide measurement range (0–200 ppm) and a higher resolution than ^1^H‐NMR. Characteristic absorption peak at 174.09 ppm, indicating that IOP‐A2 contains uronic acid, corresponding to the ‐COOH residue at the GalA (C‐6) position; two distinct signals between 90 and 110 ppm, indicating IOP‐A2 has two types of anomeric carbons, the heterocarbons of Gal and GalA. Two signal peaks at 95–105 ppm, indicating the presence of β‐galactopyranosyl and β‐galacturonic acid in IOP‐A2. There are 4 carbon signal peaks in the range of 50–80 ppm, and no signal peaks in the range of 82–88 ppm, indicating that C‐2 to C‐5 are pyran‐type carbocyclic rings. In addition, the signal at 69.5 ppm indicated that C‐6 was replaced by glucose and mannose, and 15.76 ppm and 15.51 ppm corresponded to the characteristic absorption peaks of Rha (Figure 1c).

### 4.3 Effects of IOP‐A on body weight and organ indexes in rats

**FIGURE 2:**
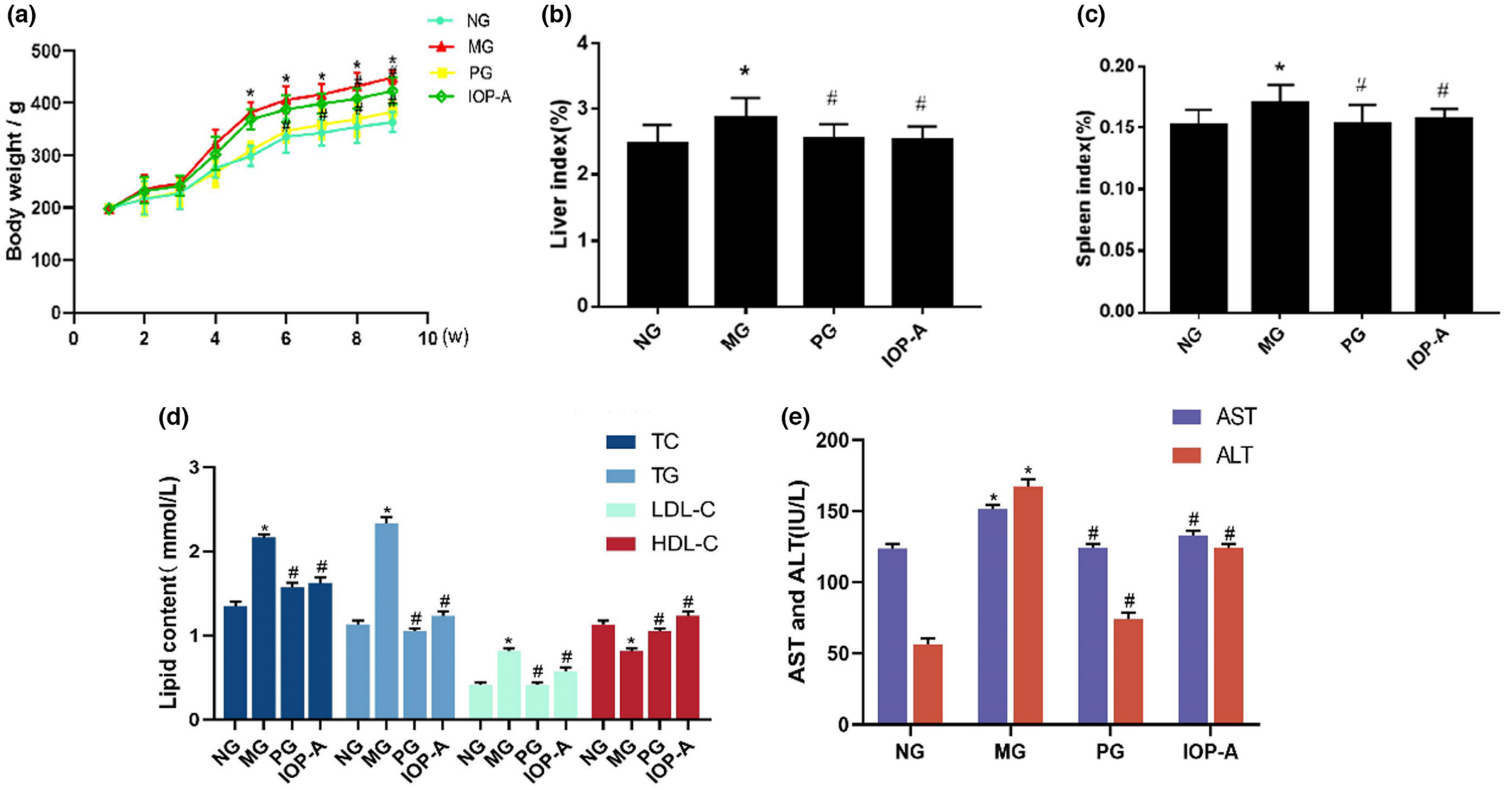
(a) Effects of IOP‐A on the body weight of rats; (b) rat liver index; (c)rat spleen index; (d) lipoprotein levels; (e) AST and ALT levels.

At the start of the experiment, the average body weight of the rats in the four different experimental groups was similar. In the untreated group (NG), the weight of the rats increased continuously, while the weight of rats fed high‐fat diets (MG) increased rapidly to the first fourth weeks, and was significantly increased to the ninth week compared with that of the rats from the NG. The growth trend of the rats treated with lovastatins (PG) was similar to that of the rats treated with IOP‐A (IOP‐A group). The weight of PG increased slowly until the ninth week and remained significantly lower than that of the rats from the MG (*p* < .05; Figure 2a).

As shown in Figure 2b, the liver and spleen indexes of the rats from the MG were significantly higher than those of the rats from the NG (*p* < .05). In contrast, the liver and spleen indexes of the rats from PG or the IOP‐A group were significantly lower than those of the rats from the MG (*p* < .05).

### 4.4 Effects of IOP‐A on serum lipoprotein levels during hyperlipidemia in rat

Serum levels of TG, TC, LDL‐C, and HDL‐C are presented in Figure 2c. The results show TC, TG, and LDL‐C levels strikingly elevated and HDL‐C levels significantly decreased in MG rats (*p* < .05). After treatment with lovastatin and IOP‐A, the levels of serum TC, TG, and LDL‐C in rats were significantly lower than those from the MG (*p* < .05), whereas the level of serum HDL‐C in rats from these two treatment groups were higher than in rats from the MG (*p* < .05). In addition, AST and ALT activity in serum Figure 2d presents the ALT and AST activities in different groups. Both AST and ALT of levels in serum were significantly elevated in MG rats than in NG rats, And PG and IOP‐A rats were significantly reduced compared with MG rats (*p* < .05; Figure 2d).

Compared with NG, *p < .05; compared with MG, ^#^ *p* < .05.

### 4.5 Effects of IOP‐A on the morpho‐pathology and the expression of CYP7A1 and SR‐B1 of liver tissues in rat during hyperlipidemia

**FIGURE 3:**
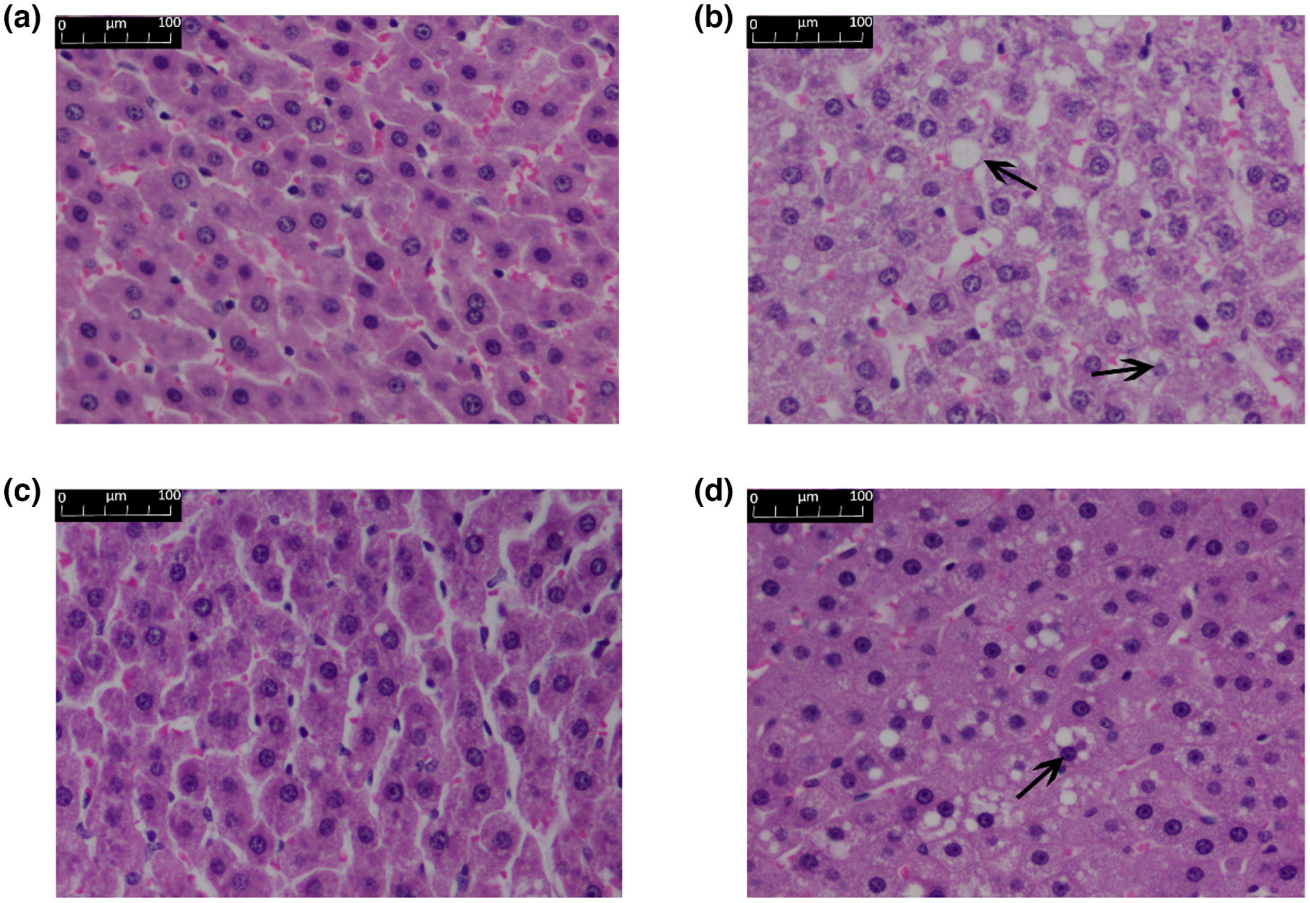
Histopathological analysis of liver sections stained with HE (400× magnification) (a) NG; (b) MG; (c) PG; (d) IOP‐A group.

In NG, histological analysis by HE staining revealed there were no fat vacuoles or fat infiltration in the hepatocytes and normal liver parenchyma with clearly defined hepatic cords (Figure 3a). In the MG, steatosis within the hepatocytes was found significantly. The hepatocytes were swollen and round with large vacuoles in the cytoplasm (Figure 3b). In contrast to MG rats, the liver pathology was significantly alleviated after lovastatin (Figure 3c) and IOP‐A (Figure 3d) treatment, as manifested by a reduced area and severity of steatosis.

**FIGURE 4:**
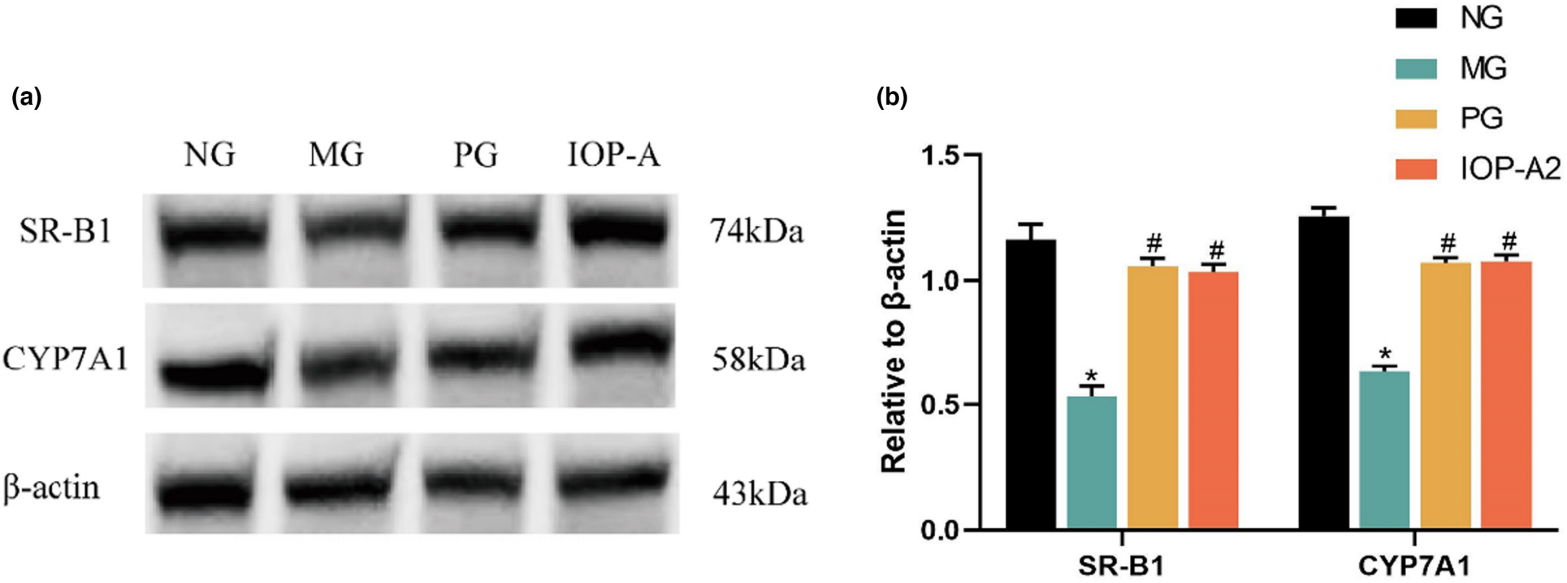
The effects of IOP‐A on expression of CYP7A1 and SR‐B1 in liver tissues. Results were presented as means ± SD (mean ± *s*, *n* = 8). Compared with NG, *p < .05; compared with MG, ^#^ *p* < .05.

The expression level of SR‐B1 and CYP7A1 proteins in the livers of MG was significantly decreased compared with those in NG (*p* < .05). In contrast, the expression level of SR‐B1 and CYP7A1 proteins in IOP‐A and PG was significantly increased, compared with that in the MG (*p* < .05; Figure 4).

### 4.6 Effects of IOP‐A2 on TC and TG contents and the expression of CYP7A1, LXRα, SR‐B1, and ABCA1 proteins in HepG2 cells

**FIGURE 5:**
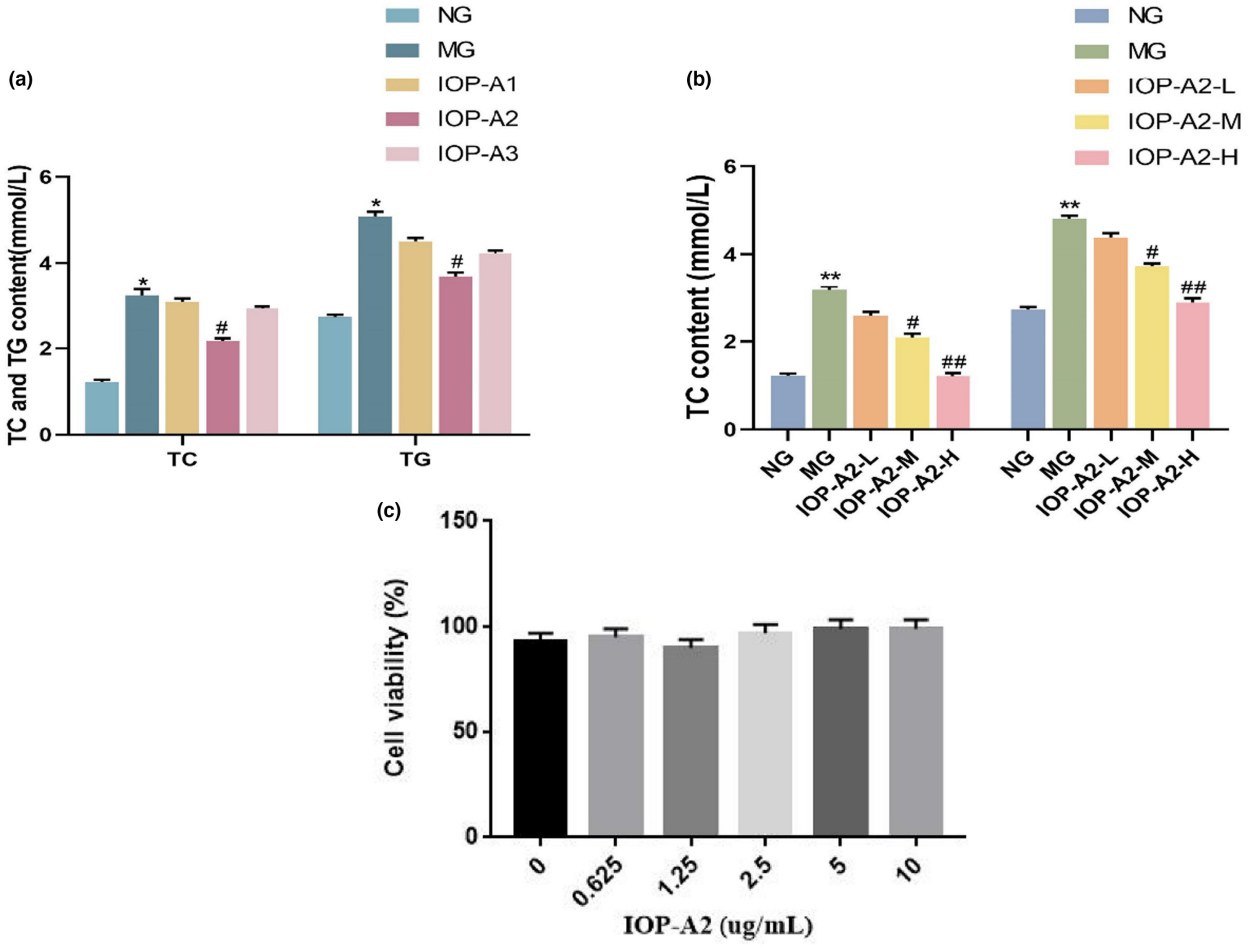
(a) Effects of IOP‐A2 on TC and TG contents in HepG2 cells induced by oleic acid (mean ± s, *n* = 6); (b) Effects of IOP‐A2 on TC and TG contents in HepG2 cells induced by oleic acid (mean ± s, *n* = 6); (c) Effects of IOP‐A2 on cell viability rate in HepG2 cells. Compared with NG, *p < .05, **p < .01; compared with MG, ^#^ *p* < .05, ^##^ *p* < .01.

As shown in Figure 5a, TC and TG content in HepG2 cells treated with IOP‐A2 decreased significantly (*p* < .05) compared with that in the NG HepG2 cells. However, no significant differences in levels of TC and TG in IOP‐A1 and IOP‐A3 groups (*p* > .05) were observed compared with those in NG cells. These results indicated that the IOP‐A2 group was shown to have hypolipidemic activity when compared to both IOP‐A1 and IOP‐A3. Thus, IOP‐A2 was selected for further investigation. As shown in Figure 5b, the TC and TG levels in the IOP‐A2‐M and the IOP‐A2‐H groups gradually decreased with the IOP‐A2 dose (*p* < 0.05 or *p* < 0.01).

In addition, the cytotoxicity of IOP‐A2 in HepG2 cells was determined using the MTT assay. 0.625–10 μg/mL LOP‐A2 was evaluated in cell experiments, as shown in Figure 5c, IOP‐A2 did not significantly affect cell viability in HepG2 cells.

**FIGURE 6:**
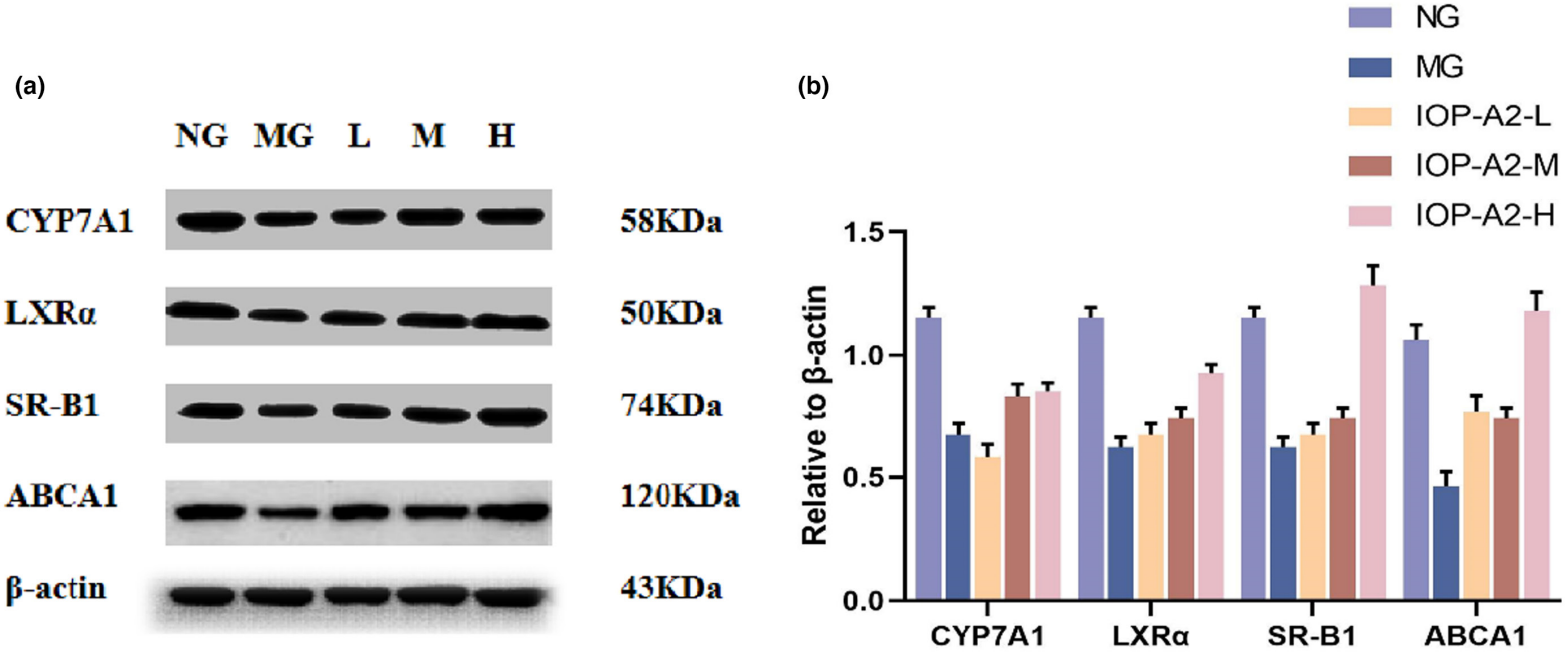
(a) The effects of IOP‐A2 on expression of CYP7A1, LXRα, SR‐B1, and ABCA1 in HepG2 cells. Results were presented as means ± SD (mean ± s, *n* = 6). Compared with NG, *p < .05; compared with MG, ^#^ *p* < .05.

The expression level of CYP7A1, LXRα, SR‐B1, and ABCA1 proteins in HepG2 cells of the MG were significantly decreased compared with that in the NG cells, (*p* < .05). Furthermore, compared with MG HepG2 cells, IOP‐A2‐H group expressed a significantly higher levels of CYP7A1, LXRα, SR‐B1, and ABCA1 proteins (*p* < .05), as shown in Figure 6.

### 4.7 Analysis of intracellular fat content by oil red O staining

**FIGURE 7:**
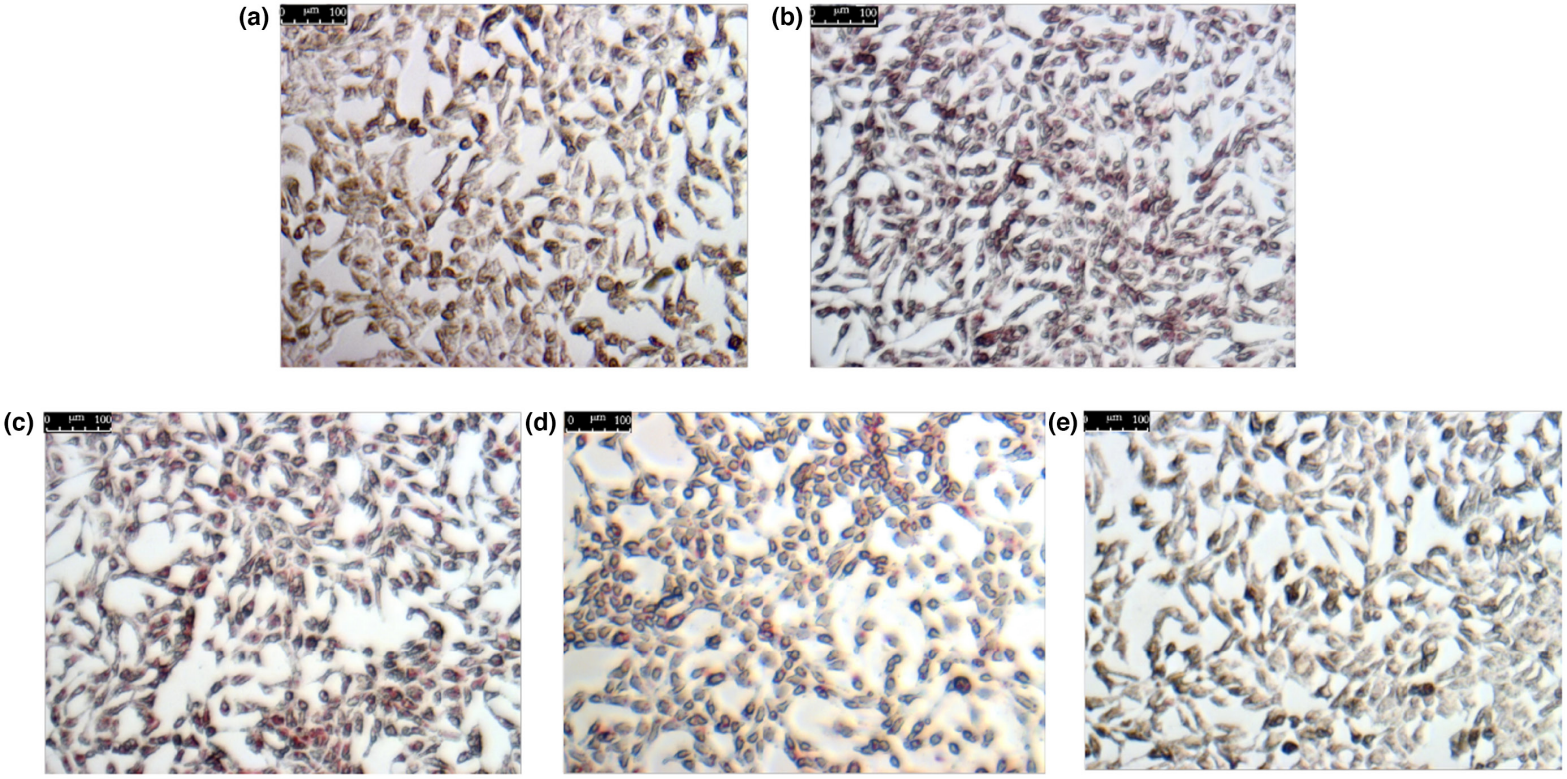
HepG2 cells stained with oil red O (400× magnification). (a) NG; (b) MG; (c) IOP‐A2‐L; (d) IOP‐A2‐M; (e) IOP‐A2‐H.

We used oil red O stain to confirm that IOP‐A2 alleviated lipid droplets compared with oleic acid‐induced HepG2 cells. HepG2 cells in the NG were ovoid with a clear‐contour shape and contained no cytoplasmic red lipid droplets (Figure 7a). In the MG group, cells exhibited heterochromatinization with accumulated cytoplasmic lipid droplets (Figure 7b). After treatment of IOP‐A2, lipid droplet accumulation decreased in a dose‐dependent manner (Figure 7c–e). And the accumulation was significantly reduced in HepG2 cells of the IOP‐A2‐H, where the lipid droplets displayed a lighter color (Figure 7e).

## 5 DISCUSSION

Hyperlipidemia is a metabolic disorder characterized by an abnormal increase of lipid components in plasma. With the improvement of living standards and the change in living habits, the incidence of it is increasing year by year. As more and more patients tend to find alternative therapies for hyperlipidemia and related cardiovascular diseases, the awareness of seeking natural products has increased among scholars. They have pointed out that some plant polysaccharides could decrease serum lipid levels (Brochot et al., 2019). Among them, IOP has a good hypolipidemic effect, but its research is not deep enough. In order to reveal the hypolipidemic effects of IOP‐A, which was obtained by hot water extraction, alcohol precipitation, deproteinization, and purification with DEAE cellulose column chromatography, a high‐fat rat model and HepG2 cell model were used to conduct in‐depth research on the hypolipidemic mechanism of IOP‐A.

It is generally known that the bioactivity of polysaccharides is associated with their molecular weight, chemical composition, glycosidic linkage, conformation and so on (Huang et al., 2016). As a result, the glycosidic linkages of polysaccharides were determined by methylation and GC–MS analysis. The main linkages in IOP‐A2 were 1,6‐Glcp, 1,3‐Glcp, 1,3,6‐Galp and 1,2‐Manp (10.59%, 8.12%, 12.60% and 9.95% respectively), and there were also a few other types of linkage. A novel water‐soluble IOP with a molecular weight of 3 kDa was first isolated, which exerted better blood lipid‐lowering activity. And the monosaccharide composition of IOP‐A consisted of Gal, Glc, GalA, Man, Rha, Xyl, GlcA, Fuc, and Ara. These findings were in agreement with other reports indicating that polysaccharides with lower molecular weights have excellent bioactivity (Fang et al., 2021; Zhao et al., 2014).

The HFD‐fed hyperlipidemic rat model has earlier been reported as an ideal in vivo model for testing antihyperlipidemic drugs (Wang et al., 2020). Using this animal model, we demonstrated the potency of using IOP‐A therapy to treat hyperlipidemia. In order to isolate and identify the lipid‐lowering components of IOP‐A, further studies are required. Three homogeneous components, IOP‐A1, IOP‐A2, and IOP‐A3, were fractionated and purified, which were screened for hypolipidemic activity using an oleic acid‐induced high steatosis model of HepG2 cells serves as a classic in vitro model for studying fatty liver disease (Wang et al., 2020). OA, easily absorbed by the cells, induces the accumulation of lipid droplets, leading to increased TC and TG cellular content (Liu et al., 2018; Xia et al., 2019; Zhang, Zhang, et al., 2019). Lipid droplets accumulated in an obvious way in HepG2 cells treated with OA, and were accompanied by increased contents of TC and TG. Treatment with IOP‐A2 significantly reduced TC and TG content in these cells, indicating that IOP‐A2 might well be the fraction of IOP‐A that mediates its hypolipidemic activity.

ALT and AST are two transaminases with wide distribution and strong activity in animals, which were the most useful tests for routine diagnosis of liver disease. Recent publications have found that ALT and AST are important biomarkers for fatty liver disease, reflecting underlying liver pathology (Sung et al., 2019). So AST and ALT were analyzed in the present study to further discuss the association between IOP‐A and its effect. Expectedly, compared to the model group, ALT and AST levels were significantly reduced in the PG and IOP‐A groups. These observations indicated that hyperlipidemia may be responsible for liver damage, and IOP‐A could attenuate liver injury following the administration.

CYP7A1 and SR‐B1 proteins are the main modulators of cholesterol metabolism and can regulate the transformation and transport of cholesterol. CYP7A1 is the first rate‐limiting enzyme during the transformation of TC into bile acid, and its transcription is positively regulated by LXRα, a member of the nuclear receptor superfamily (Lai et al., 2020; Xiao et al., 2021). When the activity of CYP7A1 decreases, the conversion of cholesterol to bile acid is blocked, resulting in an increased level of cholesterol (Zhou et al., 2021). SR‐B1 can change the distribution of cholesterol on the cell membranes, thereby mediating the uptake and reverse transport of cholesterol, and facilitating the efflux of cholesterol (Li, Sun, et al., 2020). The results of the current study revealed that IOP‐A could increase the expression of CYP7A1 and SR‐B1 proteins in the liver of diet‐induced hyperlipidemic rats. Therefore, the hypolipidemic activity of IOP‐A may be related to its capacity to regulate the metabolism and reverse transport of cholesterol.

LXRα, known as the cholesterol receptor, can be activated by specific cholesterol oxidation derivatives, such as 22 (R)‐hydroxylated cholesterol and 24 (S)‐hydroxylated cholesterol, both regulating reverse cholesterol transport (RCT) (Shi et al., 2019; Yin et al., 2019). The fact that the expression of LXRα increased significantly upon IOP‐A2 treatment suggests that IOP‐A2 may play a lipid‐lowering role by regulating RCT. During RCT, TC flows out of the cells through extrahepatic tissues and macrophages. After uptake by HDL, TC binds to ABCA1 on the cell surface, and then the HDL releases the cholesterol which forms a cholesteryl ester under the action of LCAT. The cholesteryl ester is transported back to the liver via SR‐B1, where it is metabolized into bile acid and is excreted out of the body (He et al., 2020; Wu et al., 2019). The fact that the expression of SR‐B1 and ABCA1 increased upon IOP‐A2 treatment indicates that IOP‐A2 could regulate the expression of CYP7A1, SR‐B1, and ABCA1, by increasing the expression of LXRα. This upregulation may promote the synthesis of cholesterol and HDL, accelerate the catabolism and reverse transport of cholesterol, and reduce the level and accumulation of cholesterol. Moreover, the present study revealed that IOP‐A2 increased the protein expression of CYP7A1 in OA‐induced HepG2 cells, which was consistent with its effects in vivo.

In this study, IOP‐A was isolated, purified, and identified, and its hypolipidemic effect was confirmed using a high‐fat rat model and HepG2 cell model. The results showed that IOP‐A could significantly reduce liver and spleen indexes, limit the fatty degeneration of the liver, lower TC, TG, LDL‐C, AST, and ALT levels, increase HDL‐C levels, and up‐regulate CYP7A1 and SR‐B1 protein expression in the liver of hyperlipidemic rats. IOP‐A2 could significantly reduce lipid accumulation and the level of TC and TG in HepG2 steatosis induced by OA, and it may play a hypolipidemic role mainly through the promotion of cholesterol transformation and regulation of cholesterol metabolism‐related proteins such as CYP7A1, LXRα, SR‐B1, and ABCA1. The study found that polysaccharides from traditional Chinese herbs play an important role in their medical applications, Polysaccharides have distinct advantages as drug delivery carriers instead of synthetic polymers. (Gong et al., 2022; Zeng et al., 2019). This study brings theoretical and experimental bases for future research on the hypolipidemic effect of IOP fractions and their mechanism, as well as for the development of new hypolipidemic agents.

## 6 CONCLUSION

IOP‐A2 is a 3 kD molecule isolated and purified from *I. obliquus* polysaccharides. It is composed of Gal (24.38%), Glc (24.22%), GalA (14.47%), Man (14.08%), Rha (6.76%), Xyl (5.32%), GlcA (4.91%), Fuc (0.71%), and Ara (0.14%), and its main linkage modes are 1,6‐Gl*cp*, 1,3‐Gl*cp*, 1,3,6‐Gal*p* and 1,2‐Man*p*. IOP‐A could effectively regulate lipid metabolism in hyperlipidemic rats, confirming that it represents a main active compound of IOP. In hyperlipidemia model, IOP‐A2 hypolipidemic role seems to occur mainly by promoting cholesterol metabolism and regulating the expression of the cholesterol metabolism‐related proteins CYP7A1, LXRα, SR‐B1, and ABCA1.

## AUTHOR CONTRIBUTIONS

**Guanwen Ding:** Conceptualization (equal); data curation (equal); formal analysis (equal); writing – original draft (equal); writing – review and editing (equal). **Xiao Guo:** Conceptualization (equal); data curation (equal); formal analysis (equal); funding acquisition (equal). **Xiao Li:** Methodology (equal); project administration (equal); validation (equal); writing – original draft (equal); writing – review and editing (equal). **Liping An:** Resources (equal); software (equal); supervision (equal); validation (equal). **Huawen Shi:** Formal analysis (equal); funding acquisition (equal); methodology (equal); writing – original draft (equal); writing – review and editing (equal).

## FUNDING INFORMATION

Natural Science Foundation of Jilin Province: YDZJ202201ZYTS287; Jilin City Science and Technology Bureau outstanding youth project: 20231113; Key research and development project of science and technology department of Jilin Province: 20180201049NY; Development and Reform Commission of Jilin Province, Grant/Award Number:2018C046‐3.

## CONFLICT OF INTEREST STATEMENT

There are no conflicts to declare.

## Data Availability

The data that support the findings of this study are available on request from the corresponding author.
